# Factors important in the correct evaluation of oral high‐risk lesions during the telehealth era

**DOI:** 10.1111/jop.13343

**Published:** 2022-08-23

**Authors:** Rakefet Czerninski, Netanel Mordekovich, John Basile

**Affiliations:** ^1^ Department of Oral Medicine, Sedation and Imaging, Hadassah Medical Center, Faculty of Dental Medicine Hebrew University of Jerusalem Jerusalem Israel; ^2^ Hadassah Medical Center, Faculty of Dental Medicine Hebrew University of Jerusalem Jerusalem Israel; ^3^ Department of Oncology and Diagnostic Sciences University of Maryland School of Dental Medicine Baltimore Maryland USA

**Keywords:** clinical image, early detection, high risk, oral cancer, telehealth

## Abstract

**Background:**

Considering that early detection of squamous cell carcinoma (SCC) improves prognosis and clinical examination is the primary detection method, we identified factors related to the clinical evaluation of oral mucosal lesions. Due to the growing role of telehealth, our study was based on clinical image evaluation.

**Subjects and Methods:**

Oral medicine specialists and dental students evaluated six images of benign, potentially malignant, or SCC lesions (18 images in total). We analyzed the role of personal factors of the examiners and the visual pathological features of the lesion upon which the participants based their evaluation.

**Results:**

One hundred thirty‐three subjects participated. Half of the benign images were correctly evaluated. On average 1.2 (±SD1.3) cancer pictures were recognized correctly and 3.66 (±SD1.42) images were considered potentially malignant. Potentially malignant lesions were correctly evaluated at an average of 4.08 (±SD1.48) images. For cancer and potentially malignant lesion images, there were significantly better results among clinicians with the worst results from the fourth‐year students. Student results correlated significantly with years of study, number of weeks spent in the oral medicine clinic, and interest in oral pathology. Consideration of lesion irregularity yielded a correct diagnosis, whereas wrong answers were based on color changes. Lesion size and margins were considered equally important.

**Conclusions:**

Using clinical images as part of the diagnostic process provides good results, though increased clinical experience for graduates and undergraduates may be necessary to improve accuracy. Therefore, emphasizing the important visual parameters of malignancy may be valuable in the current telehealth era.

## 1 INTRODUCTION

Clinical examination is the recommended method for detection of squamous cell carcinoma (SCC) and high‐risk oral lesions.^1^ Early detection is a major factor for improved prognosis,^2^ and is based on visual and tactile features of the lesion, symptoms, and patient history. Because the ability to recognize potentially malignant disorders or SCC is important, we wanted to study the factors affecting correct evaluation.

Potentially malignant disorders include a variety of pathologies with distinctive manifestations, so their clinical appearance is an important factor in their identification.^3^ Furthermore, follow‐up and treatment of oral mucosal pathologies rely on visualization of changes. Therefore, we analyzed factors related to the clinical evaluation of benign and high‐risk lesions, based on visual parameters. These parameters are also helpful in clinical photography, a tool recommended for the management of oral dysplasia^4^ that can monitor changes in lesions over time. Despite legal and ethical issues,^5^ sharing of clinical images has become a common consultation method between patients and clinicians and between professionals^6^ with the COVID‐19 pandemic accelerating its use.^2^, ^6^, ^7^, ^8^

A thorough clinical examination is still the gold standard for primary differential diagnosis but evaluation of clinical images is an important additional aid which is becoming more and more common. Therefore, here our first aim was to analyze factors about the examiner associated with the correct evaluation of visual features of oral lesions. The second aim was to investigate image assessment in order to determine which visual factors affect correct evaluation. Our final aim was to use our analysis to suggest ways to improve accuracy of diagnosing lesions based on clinical images.

## 2 METHODS

In order to eliminate bias, participants examined clinical images without any accompanying details and completed a questionnaire.

### 2.1 Participants

Oral medicine clinicians and dental students at the Hebrew University‐Hadassah School of Dental Medicine, in their final 3 years of study, participated. Participants were invited by email and those consenting to joining the study were sent a link to an anonymous questionnaire associated with clinical images viewed at a screen.

### 2.2 Questionnaire and images

**FIGURE A1:**
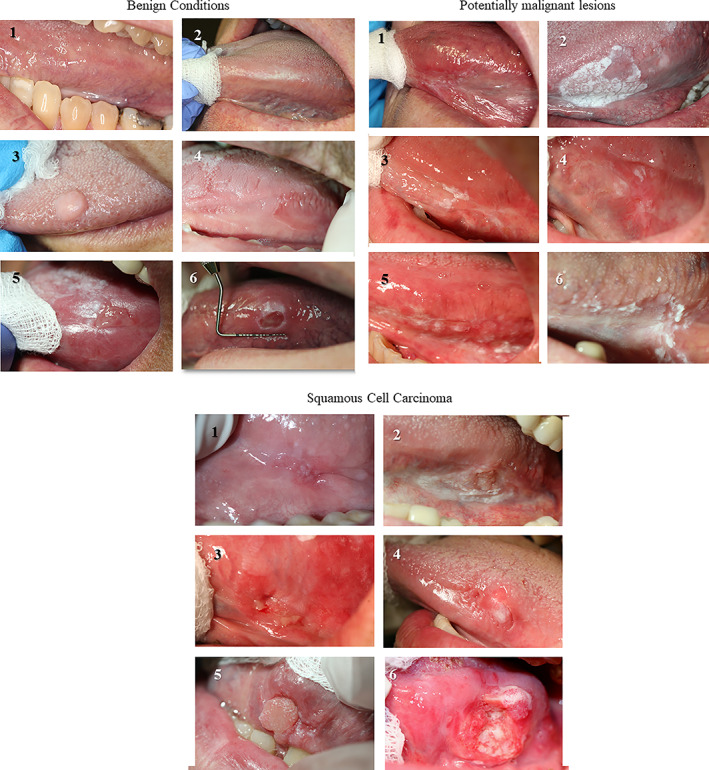
Clinical images used in the study, grouped by diagnosis: Benign conditions: (1) no changes, (2) frictional keratosis, (3) fibroma, (4) geographic tongue, (5) erosive lichen planus, (6) traumatic ulcer. Potentially malignant lesions and squamous cell carcinoma, 1–6 diagnosed by histopathology.

Images—In order to minimize variables such as site differences, we focused on the lateral tongue, which has the highest prevalence of SCC. The images used were original, taken at the oral diseases' clinic using a Canon 1200D (105/2.8 macro lens, aperture F18, shutter speed 1/80), using a ring flash. Images were 5–7 MB in size and saved as JPEGs. The pathology, when present, was located in the center of the image and in focus. Images with multiple pathologies, blurred images, and those with too bright a flash were excluded. The benign group included images of normal appearing mucosa from common, benign conditions (Figure A1). The potentially malignant disorders and malignant disorders groups included leukoplakia and erythroleukoplakia (which were diagnosed with epithelial dysplasia by biopsy) and SCC (confirmed by biopsy). These images included indurated or exophytic lesions with color and size variability (Figure A1).

A pilot study with 24 clinical images showed that the time needed for full completion of the questionnaire was approximately 20 min, with reduced attention at the last images, while 18 images allowed better attention and full compliance. The 18 original clinical images of the lateral tongue were presented in the same randomized order to all participants with no additional information. For each image the participants chose the most probable diagnosis (benign, potentially malignant, or malignant) and were asked to select which two features assisted them the most in their evaluations: size, color, margins, irregularity which refers to nonhomogenous features of the lesion (such as variation in color and warty or verrucous appearance), or lesion surface appearance (which includes more homogenous lesions demonstrating surface fissures, cracks, or nodules). We divided the responders into three groups according to their evaluation accuracy. Those evaluating up to eight images correctly (a correct answer rate of less than 50%), those with a correct evaluation of 9–11 images (51%–66%) and those who were correct for 12–18 images (67%–100%). We then investigated whether the degree of evaluation accuracy was related to any of the personal characteristics recorded: age and gender, and for students their year of study, their degree of interest in the field of oral medicine (1–10 scale, with 10 the highest interest), and the number of mandatory rotations in oral medicine clinic during their undergraduate dental training. For clinicians, the number of years of experience as a clinician was recorded.

### 2.3 Ethics

The work described is in accordance with The Code of Ethics of the World Medical Association (Declaration of Helsinki) for experiments involving humans. The study was approved by the institutional ethics committee (#30102017). Participation was voluntary, with informed consent.

### 2.4 Statistical analysis

Student's *t*‐tests or Mann–Whitney tests for nonparametric data were performed to compare quantitative variables between two groups. For comparing quantitative variables between three or more groups, analysis of variance (ANOVA) test or Kruskal–Wallis for nonparametric data were performed. If significance was found, further paired comparisons and Scheffe post hoc analysis was performed. Nonparametric tests were used when one of the groups was small and the distribution was not normal. Normality was tested by Kolmogorov–Smirnov test. In order to determine the correlation between two qualitative variables, Chi‐squared test and Fisher's exact test were used. Furthermore, in order to determine significance of a trend for a qualitative variable the linear‐by‐linear test was used. Statistical significance was set as *p* < 0.05. Data was analyzed using SPSS‐IBM version 25.

## 3 RESULTS

A total of 133 individuals participated in the study—125 students (94%) and 8 clinicians (6%). There were 50 males (37.6%) and 83 females (62.4%), aged 21–44 years with an average age of 27 years. The distribution of the year of study of the students was approximately equal for each year (Table 1). The average degree of interest in oral mucosal pathologies among students was 6.55 on a 1–10 scale. Among the clinicians, the average years of experience was 10.

**TABLE 1:** Students distribution of grade and time spent in clinical rotation.

Students	Category	*N*	%
Grade	4th	39	31.2
	5th	44	35.2
	6th	42	33.6
	Total	125	100
Number of oral medicine rotation weeks	0	45	36
	1	27	21.6
	2	53	42.4
	Total	125	100

## 4 BENIGN IMAGE EVALUATION

**FIGURE 1:**
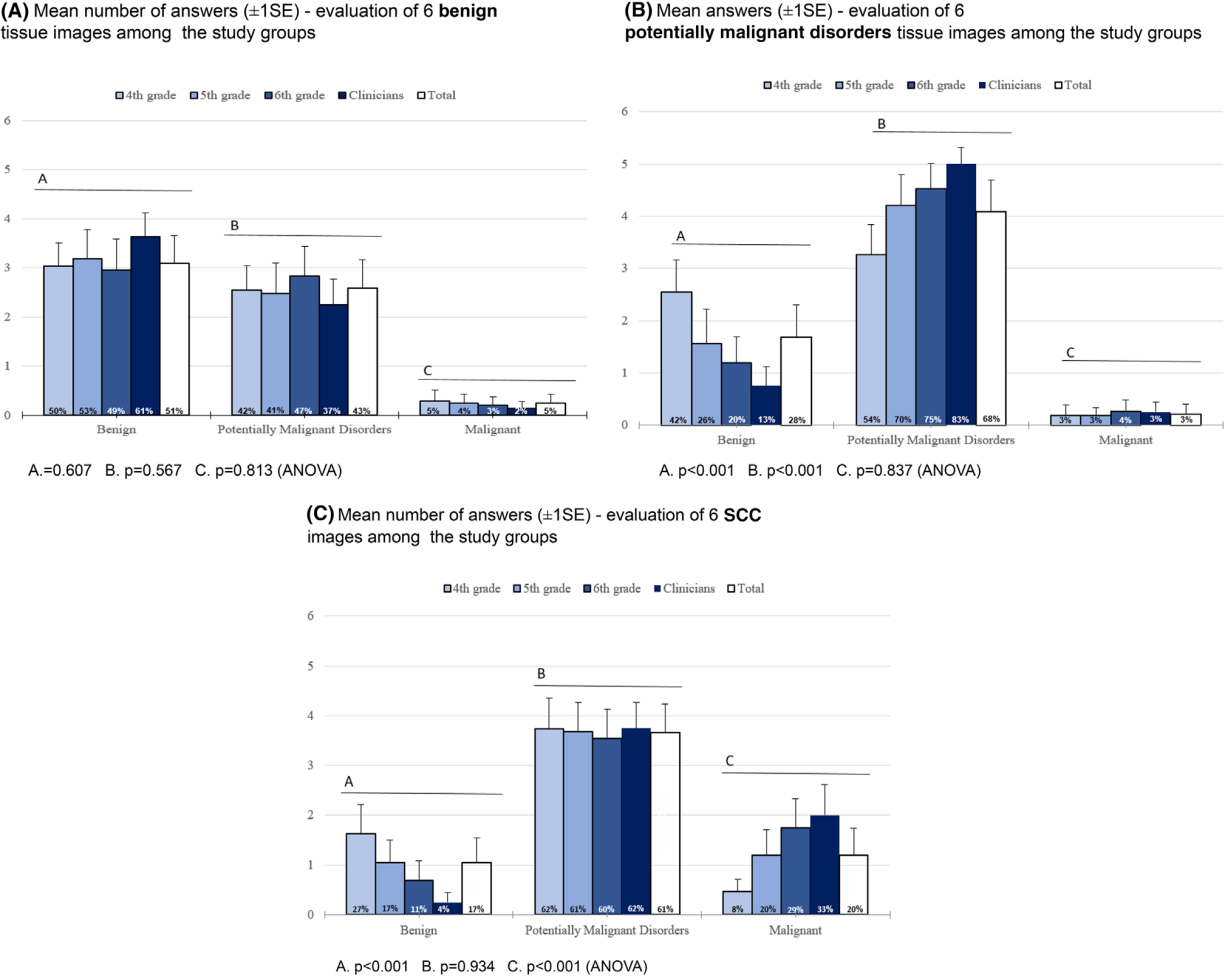
(A) The mean number of answers of the total group evaluating the images as benign were three (out of six), almost half of the pictures were evaluated as potentially malignant and only a small portion were evaluated as malignant. This pattern was also found when analyzing the answers by subgroup (clinicians and year of study). (A) *p* = 0.607; (B) *p* = 0.567; (C) *p* = 0.813 (ANOVA). (B) A mean of 4.08 images (±SD1.48) were evaluated as potentially malignant, 1.68 were evaluated as benign and 0.21 images were evaluated as malignant. A statistically significant difference was found between the evaluation of the images as potentially malignant or benign. Post hoc analysis showed that the responses of the fourth‐year students were the source of this difference. (A) *p* < 0.001; (B) *p* < 0.001; (C) *p* = 0.837 (ANOVA). (C) A mean of 1.2 (±SD1.3) of malignant lesions were evaluated correctly. Most of the pictures (mean 3.66, ±SD1.42) were evaluated as potentially malignant and the remainder as benign. In all subgroups, most of the malignant images were evaluated as potentially malignant. There was a statistically significant difference between the subgroups when comparing the mean answers evaluating the images as malignant C. (A) *p* < 0.001; (B) *p* = 0.934; (C) *p* < 0.001 (ANOVA). Post hoc analysis showed that the responses of the fourth‐year students were the source of this difference.

Analysis of the evaluations of the six benign images can be found in Figure 1A. Half the images were evaluated correctly. The evaluation pattern of the subgroups was similar, with no statistically significant difference between them (Figure 1A). *p* Values using ANOVA test were 0.607 for groups evaluating the images correctly as normal mucosa, 0.567 for the groups evaluating the images as potentially malignant disorders, and *p* = 0.813 for the groups evaluating the images as SCC.

### 4.1 Potentially malignant disorders evaluation

Analysis of the groups evaluating the six images of erythro/leukoplakia with diagnosed dysplasia is presented in Figure 1B. A mean of 4.08 images (±SD1.48) were evaluated as potentially malignant. A statistically significant difference was found between the evaluation of the images as potentially malignant (*p* < 0.001) or benign (*p* < 0.001). Post hoc analysis showed that the source of the difference was the fourth‐year students (data not shown).

## 5 MALIGNANT LESION IMAGE EVALUATION

Analysis of the groups evaluating the malignancies is presented in Figure 1C. A mean of 1.2 (±SD1.3) of the images were evaluated correctly. There was a statistically significant difference between the subgroups when comparing the mean answers evaluating the images as malignant (*p* < 0.01) or benign (*p* < 0.01) (ANOVA). Post hoc analysis showed that the responses of the fourth‐year students were the source of this difference (data not shown).

## 6 IMAGE EVALUATION AND PERSONAL CHARACTERISTICS

We divided the responders into three groups according to their evaluation accuracy and investigated whether the degree of accuracy was related to personal characteristics recorded. Table 2 presents the distribution of the accuracy results and personal characteristics and demonstrates a higher percentage of correct evaluations with more years of study, with practicing oral medicine clinicians having the greatest accuracy. This difference was statistically significant (Fisher test *p* < 0.001) even when analyzed by linear‐by‐linear association (*p* < 0.001). Table 2 also shows that those who spent at least 1‐week in the department had more correct answers. This difference was statistically significant (Fisher test *p* < 0.001) and the trend remained significant when analyzed by linear‐by‐linear association (*p* < 0.001). Finally, there was a significant trend toward more correct answers (Kruskal–Wallis test [*p* = 0.027]) for students who expressed a greater interest in mucosal pathologies (Table 2). Post hoc tests (Mann–Whitney) with the Bonferroni correction of the significance level showed that the group with lowest correct results (correct 1–8 images) was significantly different from the group with highest results (correct 12–18 images). Gender analysis showed a statistically significant difference between men and women (Pearson Chi‐squared test, asymptotic significance [two‐sided] = 0.047), with men giving more correct answers. The three groups had a similar average age.

**TABLE 2:** Distribution of correct image evaluations for all images.

		Number of correct images evaluations for all images				
		Correct 1–8 images *N* (%)	Correct 9–11 images *N* (%)	Correct 12–18 images *N* (%)	Total *N* (%)	Statistics
Grade students	4th year	32 (82.1)	7 (17.9)	0 (0)	39 (100)	*p* < 0.001^a^
	5th year	20 (45.5)	18 (40.9)	6 (13.6)	44 (100)	*p* < 0.001^b^
	6th year	18 (42.9)	17 (40.5)	7 (16.7)	42 (100)	
Clinician		0 (0)	6 (75.0)	2 (25.0)	8 (100)	
Students—Number of rotation weeks^c^	0	36 (80.0)	8 (17.8)	1 (2.2)	45 (100)	*p* < 0.001^a^
	1	14 (51.9)	11 (40.7)	2 (7.4)	27 (100)	*p* < 0.001^b^
	2	20 (37.7)	23 (43.4)	10 (18.9)	53 (100)	
Students' interest	Mean	6.11	6.95	7.62	125	0.027^d^ ^,^ ^e^
	Median	6	7	8		
	SD	2.46	2.66	2.81		
		*N* = 70	*N* = 42	*N* = 13		
Gender	Male	24 (48.0)	16 (32.0)	10 (20)^c^	50 (100)	*p* = 0.047^f^

*Note*: Responders were divided into three groups according to evaluation accuracy. Those evaluating up to eight images correctly (a correct answer rate of less than 50%), those with a correct evaluation of 9–11 images (51%–66% of images) and those who were correct for 12–18 images (67%–100%).

^a^ Fisher.

^b^ Linear by linear association.

^c^ In this study, some of students in the same class had completed 2 weeks in oral medicine, whereas others had only 1‐week or had not done any rotations.

^d^ Kruskal–Wallis test.

^e^ Post hoc tests (Mann–Whitney) with the Bonferroni correction of the significance level showed that the first group with lowest results (correct 1–8 images) was significantly different from the group with highest results (correct 12–18 images).

^f^ Pearson Chi‐square.

## 7 MAIN VISUAL FEATURES AFFECTING EVALUATION OF MALIGNANCY

The six SCC images were evaluated by 133 participants and yielded 798 responses. After deleting 11 partial responses, from the remaining 787 we analyzed which features were important to those making the correct evaluation compared to those who were incorrect (Table 3). In both groups, correct and incorrect, “lesion surface appearance” was the most important feature, and “lesion size” the least. There were no significant differences between the groups for these parameters. There was also no significant difference regarding the importance of “lesion margins.” For those with a correct evaluation, “lesion irregularity” was the second most important parameter whereas those making an incorrect evaluation did not consider this as important, with the difference between the groups being statistically significant. The group with the most incorrect responses considered “lesion color” as an important parameter, unlike those responding correctly. These results were also statistically significant (Table 3).

**TABLE 3:** Distribution of visual features affecting image evaluation.

Lesion's visual feature	Importance of the visual feature for evaluation	Number of responses who considered each visual feature as important for evaluation		*p* Pearson Chi‐square
		Wrong evaluation *N* = 771	Correct evaluation *N* = 160	
Size	**Important**	**119**	**20**	
	Not important	508	140	
Color	**Important**	**236**	**35**	***p* < 0.001**
	Not important	391	125	
Margin	**Important**	**238**	**65**	
	Not important	389	95	
Surface appearance	**Important**	**355**	**100**	
	Not important	272	60	
Irregularity	**Important**	**228**	**83**	***p* < 0.001**
	Not important	399	77	

## 8 DISCUSSION

The first aim of this study was to analyze what personal factors are associated with the correct evaluation of visual features of oral lesions. In order to reduce confounders, images were from the lateral tongue, the intraoral site with the highest prevalence of potentially malignant and malignant lesions.^9^, ^10^

We also included students in their clinical years of dental school as well as clinicians, enabling an analysis of the effects of other factors, such as experience and interest in mucosal pathology, on the evaluation.

Half of the images of the benign lesions were evaluated correctly, and almost half as pathologic (nonmalignant) lesions. There was no significant difference between the subgroups (clinicians versus students in each year). These results may be due to a high level of suspicion among the participants in a research project, making them evaluate benign images as pathologic. Clinically, a higher level of suspicion is preferable, such that this finding can be considered acceptable.

At the other end of the pathological spectrum were the images of SCC of the tongue. The clinicians had the highest number of correct evaluations followed by sixth‐year students, with the fourth‐year students showing the least accuracy. All students were already exposed to the relevant courses covering oral examination and oral potentially malignant disorders, so the differences in accuracy between fourth‐year students and the higher years shows that even a relatively short amount of clinical time improves diagnostic skills. Most SCC images were considered as pathologic and not normal by all participants. While a mistaken evaluation of SCC as potentially malignant lesion is not ideal, it usually means that the correct steps for a diagnosis (such as referral for a biopsy) will be taken. These findings reflect a clinical dilemma in differentiating between SCC and a potentially malignant lesion, which have nonspecific and overlapping visual features.^9^, ^11^, ^12^, ^13^, ^14^ While benign tissue lesions have more “normal” or innocent visual features and malignant are more “severe,” dysplastic lesions are somewhere “in the middle.”^11^ Thus, evaluations of erythro/leukoplakia images yielded better results than the malignant images, because more participants evaluated the images as pathological. When analyzing the range of correct answers, the smallest range was seen among clinicians, which implies consistency and a more precise evaluation, followed by the sixth‐year students. This trend, which is similar to that of the malignant photos, can be explained by different years of clinical experience among the subgroups. Higher clinical experience can also explain the findings of Patel et al. who reported better accuracy in diagnosing malignant or premalignant lesions among specialists compared to GP's in their in‐person evaluation of patients.^15^ Experience and training were also emphasized in Allen's study, which noted that lack of training and confidence was the most prevalent barrier to oral mucosal screening by dentists.^16^ Clinician experience was an important factor in Brocklehurst's research which studied the factors influencing practitioners' detection and the decision to refer oral lesions.^17^ Students' experience was discussed by Hassona et al. who wrote that “significant positive correlation was found between knowledge scores and early detection practice scores,” and that “students contact with patients who have oral lesions, including oral cancer will help to improve their future diagnostic ability and early detection practices.”^18^

When dividing the responders into groups based on evaluation accuracy, we found that clinicians had the best results and fourth‐year students had the poorest, following a pattern where the percentage of correct evaluations increased with experience. The relationship between better performance and more rotation weeks indicates that time spent in the oral medicine clinic improves diagnostic skills. Therefore, undergraduate programs should include at least 2 weeks of exposure to “oral medicine”‐like clinics in which nondental pathologies are examined and diagnosed. Indeed, the need for further education is also supported by the findings of others.^13^, ^19^ This point was also made at the consensus undergraduate curriculum in Oral Medicine in the United Kingdom and Republic of Ireland, where the importance of management of mucosal pathologies as a part of oral health care was discussed.^20^, ^21^ Similarly, other studies concluded that more contact with patients with oral pathologies are needed.^22^, ^23^

The next factor we wanted to examine was the amount of interest participants had in oral mucosal pathologies. The trend noted in the current study was more evaluation accuracy with greater interest, with the group reporting the highest interest having the most correct responses and vice versa. The tendency of better results with higher consistency and absolute agreement among oral medicine specialists compared to other dentists evaluating clinical images has been reported previously.^24^ Together with the current findings, these results emphasize the importance of interest and experience in visualizing oral mucosal pathologies.

Analysis of the results for all the images showed that males had higher percentage of correct answers than females. This finding was not apparent in the analysis of the subgroup of cases. Studies on gender differences in diagnostic ability have yielded inconsistent results.^13^

The importance of image evaluation and visual parameters has increased remarkably with the telehealth trend due to the COVID‐19 pandemic. In order to determine which visual features assisted in making a correct evaluation, the participants were asked to identify the main visual feature that affected their decision. The significant differences identified between correct and incorrect evaluations of SCC images were lesion irregularity and color, respectively. While all responders considered the surface appearance as a major parameter, irregularity was the feature that correct responders relied on more than the incorrect responders, suggesting its importance in rendering an accurate evaluation at the time of diagnosis. Although “irregularity” is a nonspecific term providing no clear description of visual features, it is an important feature that can reflect epithelial and subepithelial changes and includes atrophic, hyperplastic, and indurated areas.^9^, ^10^, ^11^ Color was the visual parameter wrongly considered important and significantly affecting incorrect evaluations.

Although the analysis of diagnostic abilities based on clinical images alone might be limited, this technique has been used previously.^13^, ^19^ Moreover, the participants in the current investigation showed good results compared to other studies based on clinical examination,^12^, ^25^ with general misdiagnosis of 41%–79% of mucosal lesions by dental practitioners.

Our results are applicable to both clinical and “digital” settings and support the positive attitude of younger clinicians toward this tool for teaching, monitoring premalignancy, and clinician communication.^26^ Haron et al. concluded that images captured using a smartphone camera can be integrated into the clinical setting for managing OPMD.^27^ The implementation of clinical images as part of the management of patients with potentially malignant disorders was recommended by Awadallah in 2018.^4^ This recommendation was implemented quickly during the COVID‐19 pandemic for communication and consultation or as a tool for follow‐ups,^28^ in particular for oral potentially malignant disorder patients,^29^ and head and neck cancer patients.^30^ Although not ideal for developing a differential diagnosis, there are some circumstances where clinical images provide an opportunity to make an accurate evaluation, particularly with more experienced clinicians. While the picture should demonstrate all the visible features of the lesion,^2^ clinicians should pay particular attention to features such as irregularity and surface changes which may imply a high‐risk lesion.

## 9 CONCLUSIONS

The clinical primary diagnosis of SCC and potentially malignant disorders improves with experience and interest in mucosal pathologies. Because increasing clinical experience improves diagnostic skills, it would be beneficial to expand clinical exposure during undergraduate training and in continuing education programs. The utilization of clinical images in the process of developing a diagnosis may be a helpful adjunct and provide accurate results when evaluated by properly trained clinicians emphasizing the important visual parameters of malignancy such as lesion irregularity. These findings may be of great value in the currently expanding telehealth era.

## CONFLICT OF INTEREST

The authors declare no conflict of interest.

## Data Availability

The data that support the findings of this study are available from the corresponding author upon reasonable request.
